# Diet, physical activity, and adiposity in children in poor and rich neighbourhoods: a cross-sectional comparison

**DOI:** 10.1186/1475-2891-6-1

**Published:** 2007-01-11

**Authors:** Anwar T Merchant, Mahshid Dehghan, Deanna Behnke-Cook, Sonia S Anand

**Affiliations:** 1 Population Health Research Institute, Hamilton ON, Canada; 2 McMaster University, Hamilton ON, Canada; 3 Hamilton Health Sciences, Hamilton, ON, Canada

## Abstract

**Background:**

Obesity in Canadian children increased three-fold in twenty years. Children living in low-income neighborhoods exercise less and are more overweight than those living in more affluent neighborhoods after accounting for family socio-economic status. Strategies to prevent obesity in children have focused on personal habits, ignoring neighborhood characteristics. It is essential to evaluate diet and physical activity patterns in relation to socio-economic conditions to understand the determinants of obesity. The objective of this pilot study was to compare diet, physical activity, and the built environment in two Hamilton area elementary schools serving socio-economically different communities.

**Methods:**

We conducted a cross-sectional study (November 2005-March 2006) in two public elementary schools in Hamilton, Ontario, School A and School B, located in low and high socioeconomic areas respectively. We assessed dietary intake, physical activity, dietary restraint, and anthropometric measures in consenting children in grades 1 and higher. From their parents we assessed family characteristics and walkability of the built environment.

**Results:**

160 children (n = 48, School A and n = 112, School B), and 156 parents (n = 43, School A and n = 113, School B) participated in this study. The parents with children at School A were less educated and had lower incomes than those at School B. The School A neighborhood was perceived to be less walkable than the School B neighborhood. Children at School A consumed more baked foods, chips, sodas, gelatin desserts, and candies and less low fat dairy, and dark bread than those at School B. Children at School A watched more television and spent more time in front of the computer than children studying at School B, but reported spending less time sitting on weekdays and weekends. Children at both schools were overweight but there was no difference in their mean BMI z-scores (School A = 0.65 versus School B = 0.81, p-value = 0.38).

**Conclusion:**

The determinants of overweight in children may be more complex than imagined. In future intervention programs researchers may consider addressing environmental factors, and customizing lifestyle interventions so that they are closer to community needs.

## Background

Obesity in children is increasing rapidly but interventions to prevent it have met with limited success [1]. Since obesity does not result from any one single factor, researchers have tested combined interventions, with several messages, as well as single interventions with a single message. Out of six long-term studies with combined dietary education and physical activity interventions, five resulted in no difference in overweight status between groups and one resulted in improvements for girls receiving the intervention, but not in boys [1]. In contrast, interventions with a single message, such as reducing television watching [2] or soda consumption [3], or increasing physical activity [4], have demonstrated an impact. The main reasons for interventions failing to show results have been hypothesized to be first, that the length of the interventions was insufficient, second, the children in the control group changed their behavior because they were being followed closely, and finally, the underlying social and environmental determinants of obesogenic behavior were not addressed [1]. However, it is also possible that the messages were diluted when combined, and all messages were not relevant in every situation. Therefore, customizing the messages to better meet community needs may increase the chances of success.

In preparation of an intervention study to prevent obesity among elementary school children in the Hamilton area, we conducted this pilot study of lifestyle characteristics and perceptions in two elementary schools in Hamilton located in socio-economically disparate neighborhoods. The objectives of this pilot study were to compare diet, physical activity, the built environment, and body weight in two Hamilton area elementary schools serving socio-economically different communities.

## Methods

Permission to conduct the study was obtained from the principals of two public elementary schools in Hamilton, Ontario, School A and School B, the Research Ethics Board of McMaster University, and Hamilton-Wentworth District School Board. All study personnel coming into contact with children received police clearance for a history of criminal charges following a record check.

### Study population

School A is located in a neighborhood with low socio-economic status (postal code L8L 6T9), and School B is situated in a high socio-economic status neighborhood (postal code L8S 1K6). All children in grades one and higher were approached, and those who provided written consent from their parents were included in the study. We asked the principals of both schools for permission to administer the child questionnaires and carry out the measurements at the respective schools. The School A principal agreed but the principal of School B only permitted us to conduct the measurements at the school. We therefore sent both the child and parent forms home at School B. In the instructions we requested the children to complete the child questionnaires. Children in grades 3 and higher were able to do so without help. In School A we helped the younger children with the questions and at School B we requested the parents to do so.

### Assessment

All children were asked to complete the child questionnaire and allow physical measurement of height, weight, waist and hip circumference. All parents were asked to complete a parent questionnaire.

### Child questionnaire

In this questionnaire we measured diet, dietary restraint, and physical activity. Before the questionnaire was administered, we pre-tested it among a group of Canadian children of similar ages and found the responses to have face validity.

*Food intake *was assessed using items from the Youth and Adolescent Questionnaire (YAQ) [5]. This diet assessment instrument was developed in a multiethnic sample of US children. Pearson correlation coefficients for reproducibility for nutrients ranged from 0.26 for protein and iron to 0.58 for calcium; for foods it ranged from 0.39 for meats to 0.57 for soda [5]. In a validation study the Pearson correlation coefficients ranged from 0.21 for sodium to 0.58 for folate, with an average correlation coefficient of 0.54 after correcting for within-person error [5]. Food intakes (continuous variable) were converted into servings per day by multiplying the average portion size by frequency of intake.

*Dietary restraint *reflects behavioural factors that control diet; it was measured using the three factor eating questionnaire (TEEQ), which measures cognitive restraint, uncontrolled eating, and emotional eating, and has been used in similar studies [6,7]. It has been adapted and validated for use in the general population and among adolescents. High scores on the cognizant restraint scale are positively correlated with intake of healthy foods such as green vegetables, and negatively correlated with the intake of unhealthy foods such as French fries and sugar [6]. We coded the responses so that a low score indicated little dietary restraint and a high score showed a high degree of dietary restraint. Then we summed all the responses to this set of questions to obtain a dietary restraint score.

*Physical activity *was evaluated using questions on TV watching, using the computer, watching movies, participation in organized sport, and time spent in play, from a previously validated questionnaire [8]. To estimate the average time (min/day) spent on various activities we multiplied the reported amount of time (min/day) spent in that activity by the number of days per week it was performed, and then divided by 5 to estimate average time spent on a typical weekday, by 2 for a typical weekend, and by 7 for a typical day of the week.

### Anthropometry

Child height, weight, and waist and hip circumferences were measured using a standardized protocol used in the past [9]. Height was measured without shoes correct to the nearest 0.1 cm using a stadiometer, and weight was measured in light clothes measured to the nearest 0.1 kg using a portable scale. Waist circumference was measured to the nearest 0.1 cm over the unclothed abdomen at the smallest diameter between the costal margin and the iliac crest (the hip), at the end of a normal expiration, by using a non-stretchable standard tape measure attached to a spring balance exerting a force of 750 g. Body mass index (BMI) was calculated by dividing the weight in kilograms by height in meters squared, and BMI z-scores (BMIZ) were computed using the Centers for Disease Control Anthropometric computer program [10].

### Parent questionnaire

In this questionnaire we ascertained household income, ethnicity, marital status, and education level of parents. Parental perception of neighbourhood built environment and walkability was assessed using a modified Neighborhood Environment Walkability Survey (NEWS) questionnaire [11]. The domains were: population density, street connectivity, land use mix (e.g. presence of shops and services), pedestrian-supportive infrastructure/facilities (e.g. sidewalks and lighting), esthetics, and safety [11,12]. The questions on the built environment were on an ordinal scale and re-coded so that a low score characterized a neighborhood that was not walkable, and a high score one that was walkable. The ordinal scores from questions in each domain of neighborhood walkability were summed up to obtain a total walkability score.

### Statistical methods

To compare differences between schools we used the t-test for continuous variables and the Cochran-Mantel-Hanszel chi-square test for categorical variables. We used servings per day to compare food intake, and minutes per day for physical activity. We used SAS Version 9 (Charlotte, N.C.) in all the analyses.

## Results

160 children (48 children from School A and 112 from School B), and 156 parents (43 parents of children from School A and 113 from School B) participated in this study. The general characteristics of the children and parents participating in this study are described in Tables 1 and 2. Briefly, responses to questions confirmed that School A parents were more socially disadvantaged than School B parents. Parents of children who studied at School A were less educated and had lower incomes than those whose children attended School B; they also had higher reported BMI (27.1 versus 23.3 kg/m^2^, p-value < 0.001).

**Table 1:** Characteristics of children

	**School A**	**School B**	**Both**
	N = 48	N = 112	N = 160
**Child**			
Age y (mean, SD)	11.2, 2.0	8.2, 1.5	9.0, 2.1
Male (%, n)	47.9%, 23	48.2%, 54	48.1%, 77
***Ethnicity***			
White (%, n)	73.0%, 33	77.5%, 79	76.2%, 112
Black (%, n)	8.9%, 4	1.0%, 1	3.4%, 5
Chinese (%, n)	0.0, 0	13.7%, 14	9.5%, 14
South Asian (%, n)	2.2%, 1	3.9%, 4	3.4%, 5
Other Asian (%, n)	6.7%, 3	2.0%, 2	3.4%, 5
Other (%, n)	8.9%, 4	2%, 2	4%, 6
***Grade***			
Grade 1 (%, n)	2.1%, 1	24.7%, 22	16.8%, 23
Grade 2 (%, n)	6.3%, 3	21.4%, 19	16.1%, 22
Grade 3 (%, n)	8.3%, 4	21.4%, 19	16.8%, 23
Grade 4 (%, n)	2.1%, 1	22.5%, 20	15.3%, 21
Grade 5 (%, n)	14.6%, 7	10.1%, 9	11.7%, 16
Grade 6 (%, n)	12.5%, 6	0.0, 0.0	4.4%, 6
Grade 7 (%, n)	29.2%, 14	0.0, 0.0	10.2%, 14
Grade 8 (%, n)	25%, 12	0.0, 0.0	8.8%, 12
BMI z-scores (mean, SD)	0.65, 1.14	0.81, 0.71	0.75, 0.88

**Table 2:** Characteristics of parents

	**School A**	**School B**	**Both**
**Parents**	N = 43	N = 113	N = 156
***Father's Education***			
High School or less (%, n)	65%, 28	13.5%, 13	29.5%, 41
Trade School (%, n)	25.6%, 11	17.7%, 17	20.1%, 28
Bachelor's degree (%, n)	0, 0	22.9%, 22	15.8%, 22
Postgraduate (%, n)	4.7%, 2	44.8%, 43	32.7%, 45
***Mother's Education***			
High School or less (%, n)	63%, 29	11%, 11	27.4%, 40
Trade School (%, n)	26.1%, 12	21%, 21.0	22.6%, 33
Bachelor's degree (%, n)	8.7%, 4	42%, 42	31.5%, 46
Postgraduate (%, n)	2.2%, 1	26%, 26	18.5%, 27
***Annual Family Income***			
< $20,000 (%, n)	13.3%, 6	9.2%, 9	10.5%, 15
$20–30,000 (%, n)	29%, 13	7.1%, 7	14%, 20
$31–45,000 (%, n)	20%, 9	7.1%, 7	11.2%, 16
$46–65,000 (%, n)	26.7%, 12	14.3%, 14	18.2%, 26
$66–90,000 (%, n)	11.1%, 5	28%, 27	22.4%, 32
>$90,000	0, 0	34.7%, 34	23.8%, 34
***Marital status of parents***			
Never Married (%, n)	8.3%, 4	2.9%, 3	4.6%, 7
Married (%, n)	64.6%, 31	83.5%, 86	77.5%, 117
Common Law (%, n)	16.7%, 8	3.9%, 4	8%, 12
Widowed, Separated, or Divorced (%, n)	10.5%, 5	9.7%, 10	10.0%, 15
***Parental BMI, kg/m***^2^***(mean, SD) ***	27.3, 6.0	23.2, 4.0	24.6, 5.1

About half the children were males at both schools; however, the children at School A were older than those at School B (11.0 vs. 8.1 years respectively, p-value < 0.001). Dietary analyses did not reveal differences in fruit, vegetable, and legume consumption among children at the two schools, although children at School A consumed more baked foods, chips, sodas, gelatin desserts, and candies, and less low fat dairy, and dark bread than children at School B (Table 3). No significant difference in dietary restraint between children at the two schools was identified (dietary restraint score was 15 for School A versus 14 for School B).

**Table 3:** Comparison of child intake of selected foods (servings/d) by school

	**School A**	**School B**	**Both**
	N = 46	N = 112	N = 158
	Mean (SD)	Mean (SD)	Mean (SD)
Green leafy vegetables *	0.25 (0.37)	0.39 (0.34)	0.35 (0.36)
Cruciferous vegetables *	0.39 (0.52)	0.23 (0.20)	0.28 (0.34)
All vegetables	2.09 (2.05)	1.90 (1.10)	1.96 (1.44)
Legume *	0.21 (0.47)	0.26 (0.33)	0.25 (0.37)
Fruit	4.78 (2.80)	4.77 (2.39)	4.78 (2.51)
Fruit and vegetables	6.71 (4.07)	6.70 (3.18)	6.71 (3.45)
Dairy *	2.02 (1.77)	2.80 (1.43)	2.60 (1.56)
Low fat dairy *	1.01 (1.05)	1.38 (0.80)	1.27 (0.89)
Juice	1.89 (1.50)	1.53 (1.04)	1.64 (1.20)
Soda *	0.74 (0.80)	0.12 (0.28)	0.30 (0.57)
Sugar drink *	2.34 (1.82)	1.59 (1.07)	1.81 (1.37)
Baked *	3.61 (3.07)	0.95 (0.77)	1.72 (2.13)
Chips *	0.64 (1.05)	0.20 (0.34)	0.32 (0.66)
Gelatin desserts *	4.16 (2.11)	0.22 (0.25)	1.35 (2.12)
Candy *	8.85 (2.95)	0.38 (0.42)	2.90 (4.22)
Cracker *	0	0.30 (0.30)	0.30 (0.30)
Peanut *	0.15 (0.25)	0.06 (0.12)	0.09 (0.17)
Dark bread *	0.39 (0.81)	0.77 (0.94)	0.66 (0.92)
White bread *	1.01 (1.13)	0.58 (0.79)	0.71 (0.93)

*p-value < 0.05 comparing the two schools using the t-test

Sedentary behavior analyses indicated that children at School A watched more television and spent more time in front of the computer than children at School B, but they reported spending less time sitting on weekdays and weekends (Table 4). As School B had better standardized test results than School A, it could be speculated that the children at School B were spending more time studying even though they were watching less television, and hence being more sedentary, than those at School A. Children at both schools were overweight but there was no difference in their mean BMI z-scores (School A = 0.65 versus School B = 0.81, p-value = 0.38).

**Table 4:** Comparisons of time children spent participating in physical activity by school

	**School A**	**School B**	**Both**
	N = 46	N = 111	N = 153
	Mean (SD)	Mean (SD)	Mean (SD)
Watching television min/d *	102 (115)	18 (56)	43 (87)
In front of computer min/d *	107 (175)	6 (45)	37 (113)
Sitting on weekdays min/d	135 (144)	268 (672)	181 (413)
Sitting on weekends min/d *	43 (51)	61 (31)	47 (47)

*p-value < 0.05 comparing the two schools using the t-test

Overall, the neighborhood in which School A is located was perceived to be less walkable than the School B neighborhood. The School A neighborhood scored lower in the domains of safety, esthetics, and population density than School B, but there was no difference in the score for presence of facilities, such as streetlights, sidewalks, and parks (Table 5).

**Table 5:** Comparisons of parental assessment of characteristics built environment by school^†^

	**School A**	**School B**	**Both**
	N = 48	N= 95	N = 143
	Mean (SD)	Mean (SD)	Mean (SD)
Safety *	21 (4)	23 (3)	23 (3)
Residential density *	14 (2)	16 (2)	15 (2)
Esthetics *	7 (2)	10 (2)	9 (2)
Presence of facilities	9 (2)	9 (2)	9 (2)

*p-value < 0.05 comparing the two schools using the t-test

^†^A higher score represents a built environment favoring walking

## Discussion

The mean BMI z-scores of the children in the two schools were similar, even though they came from different socio-economic backgrounds, ate different foods, and had different physical activity patterns. School A households had lower parental income and education levels than School B households. The School A neighborhood was perceived as being less walkable than the School B neighborhood. Children at School A ate more junk food but were more active than those at School B. The factors contributing to body weight of children in these two schools were likely different.

Our findings are inconsistent with prior studies that demonstrate the powerful influence of the environment on obesogenic lifestyles. Canadian children living in neighborhoods with low mean income were more likely to be overweight or obese compared with those living in neighborhoods with high mean income, after accounting for family income and individual characteristics [13,14]. Janssen et al reported that Canadian adolescents living in low-income neighborhoods were more likely to be obese after accounting for family affluence, perceived family affluence, age, and sex in a large national sample [13].

These results imply that some characteristics of the neighborhood predispose children to obesity independent of demographic and socio-economic factors. The foods available in low-income neighborhoods are of lower quality [15], cost more, and have less variety, than foods available in more affluent neighborhoods, because larger suppliers tend to target higher income consumers [16]. Moreover, healthy foods such as fruits and vegetables, poultry, fish and whole grain cost more compared with less healthy alternatives which may promote obesity such as refined grain, French fries, bakery products, and snacks containing high sugar and fat [17]. Likewise, low-income neighborhoods have fewer facilities for recreational physical activity; the presence of facilities in neighborhoods is directly correlated with individual physical activity and BMI [18].

Our study had some limitations. First, the sample size was small, which increased the likelihood of type 2 error (power = 14%, with alpha level of 0.05). Non-significant results should therefore be interpreted with caution. A second limitation was that these children were self-selected, and may have been more motivated and health conscious than the general population. This may explain why fruit and vegetable intake among children at both schools was high. Third, the children in School A were older than those in School B, and may be one reason why junk food intake was higher at School A. Fourth, information on diet and physical activity were obtained from self-report and could result in biased reporting. Last, the way data were collected at the two schools was different. Children at School B filled the diet and physical activity questionnaires at home and may have reported more healthy behaviors because of parental influence, while those at School A completed the questionnaires at school. However, even though total vegetable intakes at the two schools were similar, there were differences in the reported intakes of types of vegetables; those at School A reported eating more cruciferous vegetables, while those at School B more green leafy vegetables. Similarly, children at School A reported being more physically active than those at School B, and there were differences in the perception of the walkability of the neighborhood reported by the parents. Taken together, these results suggest that reporting bias was probably not a large factor in the study. However, in a larger investigation, these limitations would need to be addressed by supplementary objective measures for physical activity such as pedometers, and biomarkers or alternative nutritional assessment for diet.

The main implication of our study is that the factors causing obesity in communities may be quite different even though they are in the same city (within 10 km of each other). Customizing messages to meet community needs may make interventions to prevent weight gain more effective. For instance, at School A the main messages may be to reduce television time, soda, and baked food consumption, while at School B it could be for the children to be more active on weekdays and weekends. There is evidence that ethnicity, family characteristics, and behavior influence physical activity [20] and obesity [21] in Canadian children, but these factors have not been adequately evaluated. Because populations are heterogeneous, one set of messages may be redundant for many of the participants, and may be an unappreciated reason for the failure to observe clear benefits in obesity prevention trials in children.

Most intervention studies to date have first, targeted the individual (and generally ignored the environment), and second promoted a standard message at all the intervention sites. Future intervention studies may therefore overcome these limitations by evaluating structural changes that are anti-obesogenic by design, and customizing their messages for target communities. These conclusions are consistent with the findings from a recent review of interventions to prevent overweight and obesity in children [19].

In future intervention programs researchers may consider addressing environmental factors that can impact obesity, and customizing lifestyle interventions so that they are closer to community needs.

## References

[B1] Summerbell CD, Waters E, Edmunds LD, Kelly S, Brown T, Campbell KJ (2005). Interventions for preventing obesity in children. Cochrane Database Syst Rev.

[B2] Robinson TN, Wilde ML, Navracruz LC, Haydel KF, Varady A (2001). Effects of reducing children's television and video game use on aggressive behavior: a randomized controlled trial. Arch Pediatr Adolesc Med.

[B3] James J, Thomas P, Cavan D, Kerr D (2004). Preventing childhood obesity by reducing consumption of carbonated drinks: cluster randomised controlled trial. BMJ.

[B4] Mo-suwan L, Pongprapai S, Junjana C, Puetpaiboon A (1998). Effects of a controlled trial of a school-based exercise program on the obesity indexes of preschool children. Am J Clin Nutr.

[B5] Rockett HR, Breitenbach M, Frazier AL, Witschi J, Wolf AM, Field AE, Colditz GA (1997). Validation of a youth/adolescent food frequency questionnaire. Prev Med.

[B6] de Lauzon B, Romon M, Deschamps V, Lafay L, Borys JM, Karlsson J, Ducimetiere P, Charles MA (2004). The Three-Factor Eating Questionnaire-R18 is able to distinguish among different eating patterns in a general population. J Nutr.

[B7] Westenhoefer J, Stunkard AJ, Pudel V (1999). Validation of the flexible and rigid control dimensions of dietary restraint. Int J Eat Disord.

[B8] Gortmaker SL, Peterson K, Wiecha J, Sobol AM, Dixit S, Fox MK, Laird N (1999). Reducing obesity via a school-based interdisciplinary intervention among youth: Planet Health. Arch Pediatr Adolesc Med.

[B9] Anand SS, Yusuf S, Vuksan V, Devanesen S, Teo KK, Montague PA, Kelemen L, Yi C, Lonn E, Gerstein H, Hegele RA (2000). Differences in risk factors, atherosclerosis and cardiovascular disease between ethnic groups in Canada: the study of health assessment and risk in ethnic groups (SHARE). Indian Heart J.

[B10] (2006). A SAS Program for the CDC Growth Charts. Centers for Disease Control.

[B11] Brownson RC, Chang JJ, Eyler AA, Ainsworth BE, Kirtland KA, Saelens BE, Sallis JF (2004). Measuring the environment for friendliness toward physical activity: a comparison of the reliability of 3 questionnaires. Am J Public Health.

[B12] Saelens BE, Sallis JF, Black JB, Chen D (2003). Neighborhood-based differences in physical activity: an environment scale evaluation. Am J Public Health.

[B13] Janssen I, Boyce WF, Simpson K, Pickett W (2006). Influence of individual- and area-level measures of socioeconomic status on obesity, unhealthy eating, and physical inactivity in Canadian adolescents. Am J Clin Nutr.

[B14] Veugelers PJ, Fitzgerald AL (2005). Prevalence of and risk factors for childhood overweight and obesity. CMAJ.

[B15] Zenk SN, Schulz AJ, Israel BA, James SA, Bao S, Wilson ML (2006). Fruit and vegetable access differs by community racial composition and socioeconomic position in Detroit, Michigan. Ethn Dis.

[B16] Moore LV, Diez Roux AV (2006). Associations of neighborhood characteristics with the location and type of food stores. Am J Public Health.

[B17] Drewnowski A, Darmon N (2005). Food choices and diet costs: an economic analysis. J Nutr.

[B18] Gordon-Larsen P, Nelson MC, Page P, Popkin BM (2006). Inequality in the built environment underlies key health disparities in physical activity and obesity. Pediatrics.

[B19] Rhodes RE, Macdonald HM, McKay HA (2006). Predicting physical activity intention and behaviour among children in a longitudinal sample. Soc Sci Med.

[B20] Doak CM, Visscher TL, Renders CM, Seidell JC (2006). The prevention of overweight and obesity in children and adolescents: a review of interventions and programmes. Obes Rev.

